# CD26/dipeptidyl peptidase IV (CD26/DPPIV) is highly expressed in peripheral blood of HIV-1 exposed uninfected Female sex workers

**DOI:** 10.1186/1743-422X-7-343

**Published:** 2010-11-25

**Authors:** Elijah M Songok, Bernard Osero, Lyle Mckinnon, Martin K Rono, Winnie Apidi, Elizabeth J Matey, Adrienne FA Meyers, Ma Luo, Joshua Kimani, Charles Wachihi, Blake T Ball, Frank A Plummer, Solomon Mpoke

**Affiliations:** 1 Centre For Virus Research, Mbagathi Road Kenya Medical Research Institute, Nairobi, Kenya; 2 Department of Medical Microbiology, University of Manitoba,742 Bannatyne Avenue, Winnipeg, MB, Canada; 3 University of Nairobi Institute of Tropical and Infectious Diseases (UNITID), Nairobi, Kenya; 4 Department of Medicine, University of Toronto, Medical Sciences Building, 1 Kings College Circle, Toronto, ON M5S 1AB, Canada; 5 National Microbiology Laboratories, Public Health Agency of Canada, 1015 Arlington Avenue, Winnipeg, MB, R3E 3RE, Canada

## Abstract

**Background:**

Design of effective vaccines against the human immunodeficiency virus (HIV-1) continues to present formidable challenges. However, individuals who are exposed HIV-1 but do not get infected may reveal correlates of protection that may inform on effective vaccine design. A preliminary gene expression analysis of HIV resistant female sex workers (HIV-R) suggested a high expression CD26/DPPIV gene. Previous studies have indicated an anti-HIV effect of high CD26/DPPIV expressing cells in vitro. Similarly, high CD26/DPPIV protein levels in vivo have been shown to be a risk factor for type 2 diabetes. We carried out a study to confirm if the high CD26/DPPIV gene expression among the HIV-R were concordant with high blood protein levels and its correlation with clinical type 2 diabetes and other perturbations in the insulin signaling pathway.

**Results:**

A quantitative CD26/DPPIV plasma analysis from 100 HIV-R, 100 HIV infected (HIV +) and 100 HIV negative controls (HIV Neg) showed a significantly elevated CD26/DPPIV concentration among the HIV-R group (mean 1315 ng/ml) than the HIV Neg (910 ng/ml) and HIV + (870 ng/ml, p < 0.001). Similarly a FACs analysis of cell associated DPPIV (CD26) revealed a higher CD26/DPPIV expression on CD4+ T-cells derived from HIV-R than from the HIV+ (90.30% vs 80.90 p = 0.002) and HIV Neg controls (90.30% vs 82.30 p < 0.001) respectively. A further comparison of the mean fluorescent intensity (MFI) of CD26/DPPIV expression showed a higher DPP4 MFI on HIV-R CD4+ T cells (median 118 vs 91 for HIV-Neg, p = 0.0003). An evaluation for hyperglycemia, did not confirm Type 2 diabetes but an impaired fasting glucose condition (5.775 mmol/L). A follow-up quantitative PCR analysis of the insulin signaling pathway genes showed a down expression of NFκB, a central mediator of the immune response and activator of HIV-1 transcription.

**Conclusion:**

HIV resistant sex workers have a high expression of CD26/DPPIV in tandem with lowered immune activation markers. This may suggest a novel role for CD26/DPPIV in protection against HIV infection in vivo.

## Background

The disease AIDS ranks as one of the most devastating scourges of mankind. Since it was identified in 1983 more than 30 million people have died and an estimated 33 million currently live with the virus [1]. The majority of HIV infections occur in Sub-Saharan Africa, where in some countries prevalence rates of more than 40% have been documented among antenatal clinic attendees [2]. Despite progress made with antiretroviral therapy, less than a half of those requiring treatment receive it and new infections far outpace those on therapy [3]. Similar to previous viral epidemics, prevention through vaccination is believed to be the best approach. However several factors including HIVs ability to integrate itself to the host genome and to constantly mutate challenge the design of a safe and effective vaccine. More critically, a sufficient understanding of the immune correlates of protection from HIV infection and disease remains unresolved.

Individuals who though highly exposed to HIV but do not get infected (HIV R) provide an opportunity to better understand what mediates HIV protection. HIV R populations have been identified among intravenous drug users,[4], children born to HIV infected mothers [5,6], discordant couples [7], and commercial sex workers [8]. Of the various investigations conducted on HIV R, the Nairobi commercial sex worker cohort has provided vital clues for the understanding of correlates for protection against HIV infection. With more than 25 years of follow-up, this population has provided compelling evidence that HIV resistance is biologically mediated. The 32 base pair deletion in the CCR5 gene (CCR5 Δ32), which has been reported in Caucasian populations to be responsible for cellular resistance to HIV, has not been observed in this cohort nor have polymorphisms in other cytokine regions reported elsewhere to be connected to HIV resistance [9]. Although cytotoxic T-cell responses and HIV specific mucosal IgA have been noted in the Nairobi cohort [10], controversy surrounds whether they exert a protective effect or reflect a prior exposure to HIV [11]. A genetic basis for HIV resistance has been implied by the finding that among sex worker relatives of the HIV-1 resistant women, there has been a high degree of interfamily correlation of HIV infection independent of the HLA status [12]. This suggests that other genes in pathways beyond the HLA cluster may be key in elucidating the HIV resistance phenomenon.

A recent preliminary gene expression analysis of HIV resistant female sex workers who had been in active sex work for periods of more than seven years and remained HIV uninfected suggested a high expression of CD26/dipeptidyl peptidase IV (CD26/DPPIV) a gene coding for a multifunctional enzyme [13]. Previous studies have shown however that difference in genetic expression might not always correspond to similar protein levels [14]. As high expression of CD26/DPPIV has been shown previously to inhibit HIV-1 infection in vitro [15] validating gene expression reports among the HIV resistant female sex workers through protein analysis may support a quest for further functional studies on CD26/DPPIV gene as a novel contributor to the HIV resistant phenotype. In addition, high CD26/DPPIV protein levels in vivo have been shown to be a risk factor for type 2 diabetes [16]. Incretin hormones involved in insulin signaling have been shown to be degraded by high CD26/DPPIV levels leading to insulin resistance and hyperglycemia.

The present study therefore was to confirm if the high expression of CD26/DPPIV reported at the genetic level among the HIV resistant sex workers were concordant with higher peripheral blood protein levels, and whether this corresponded to clinical type 2 diabetes and perturbation of other genetic events related to insulin signaling.

## Results

**Figure 1:**
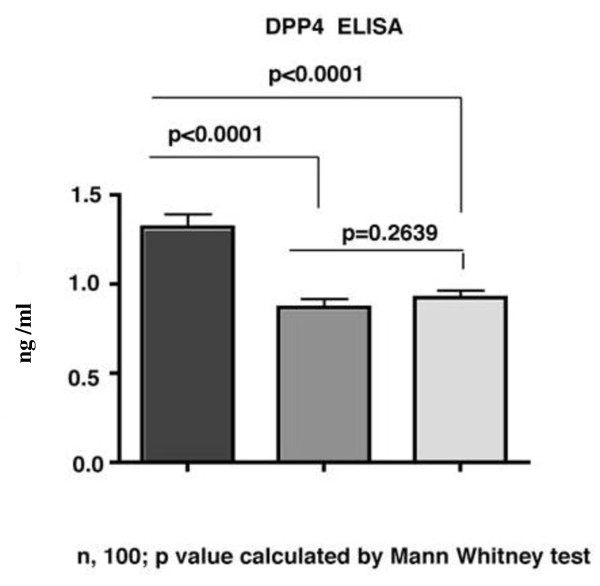
**Plasma concentrations of Dipeptidyl peptidase IV enzyme among HIV resistant (HIV-R.) HIV positive (HIV+.) and HIV negative (HIV neg.) controls (n = 100, each).ELISA (*R&D Systems, Minneapolis MN*), was done in duplicate: dilution factor 1:1000**.

To investigate the level of expression of CD26/dipeptidyl peptidase IV among HIV highly exposed uninfected sex workers, we conducted a quantitative DPPIV ELISA on plasma samples obtained from 100 HIV resistant sex workers (HIV-R), 100 HIV infected (HIV+) and 100 HIV negative controls (HIV Neg). CD26/DPPIV plasma concentration was significantly elevated among the HIV-R group (mean 1315 ng/ml) than the HIV Neg (910 ng/ml) and HIV + (870 ng/ml, p < 0.001, Figure 1).

**Figure 2:**
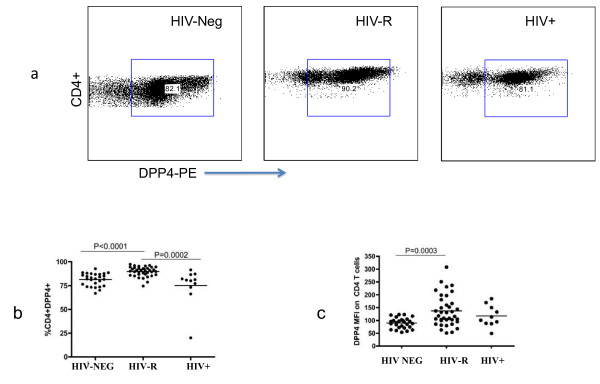
**Increased CD26/DPPIV expression on CD4+ T cells from HIV-R subjects (n=80)**.  **A)** Representative flow cytometry plots showing DPP4 expression gated on  CD4+ T cell; **B)** HIV-R have higher expression levels than HIV+ and HIV  Neg; **C)** HIV-R have higher DPP4 mean florecent intensity than HIV Neg.

To determine whether the observed differences in soluble plasma expression would correspond to differences CD26/DPPIV expression on T-cells we carried out a FACs analysis on peripheral mononuclear cells derived from the HIV-R, HIV-Neg and HIV+ women. These data revealed higher CD26/DPPIV expression on CD4+ T-cells from the HIV-R group when compared to HIV+ (90.30% vs 80.90 p = 0.002) and HIV Neg controls (90.30% vs 82.30 p < 0.001) respectively (Figure 2a). No difference in CD26/DPPIV expression on CD8+ T cells was observed between the groups (Data not shown). To further characterise CD26/DPPIV expression on CD4+ T-cells, we compared the mean fluorescent intensity (MFI) of CD26/DPPIV expression between HIV-R, HIV Neg and HIV+. This analysis showed a higher CD26/DPPIV MFI on resistant CD4+ T cells compared to HIV-Neg (median 118 vs 91, p = 0.0003, Figure 2b) suggesting that more CD26/DPPIV molecules are expressed per CD4+T cell in HIV-R subjects.

**Figure 3:**
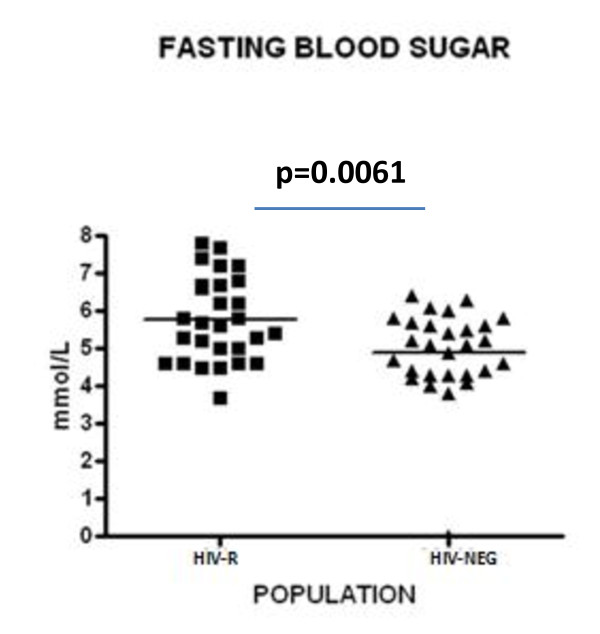
**Fasting Blood sugar levels of HIV Resistant (HIV-R) and HIV negative controls (HIV-Neg),(n = 27 each)**. Blood samples were taken and tested using the glucose oxidase test system (Accu-check, Roche, Basel).

As high CD26/DPPIV expression has been associated with type 2 diabetes [16], we next determined the blood glucose level of HIV-R women (mean age 38.5 ± 5). Their mean fasting blood sugar level was 5.775 mMol/L (103 mg/dL, Figure 3). Although this was significantly higher than the HIV-Neg (4.886 mmol/L or 87.94 mg/dL p = 0.0061) this was not indicative of clinical Type 2 diabetes mellitus but suggested a prediabetic or impaired fasting glucose state. The 103 mg/dL of the mean fasting blood sugar level of HIV-R subjects was lower than the WHO criteria (110 mg/dL) and slightly higher than the ADA criteria (100 mg/dL) for the impaired fasting glucose state [17,18].

**Figure 4:**
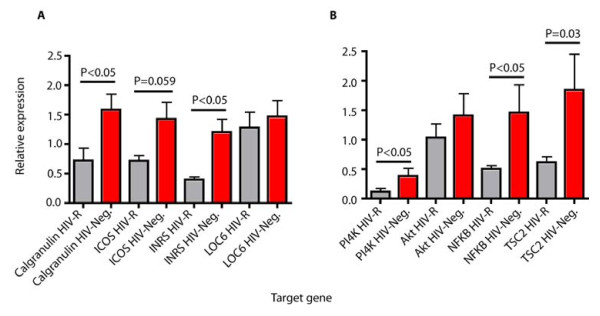
**Quantitative RT PCR expression patterns of genes previously associated with Type 2 diabetes and in the insulin signaling pathway between HIV Resistant (HIV -R and HIV negative controls (HIV-Neg) (n = 6 each)**. Calg-Calgranulin, ICOS-Inducible T-cell costimulator, INRS-Insulin receptor substrate 1, LOC6-LOC6 gene, PI4K-phospatidylinositol 4 kinase, Akt-serine/threonine protein kinase, NFkB-nuclear factor kappa B protein, TSC2-Tuberin.

To elucidate further the relationship between HIV-R female sex workers with the diabetic or prediabetic phenotype, we investigated the expression patterns of genes in the insulin signaling pathway which are significantly perturbed during Type 2 diabetes state. To achieve this we randomly selected six HIV -R and an equal number of HIV-Neg controls from the study population. We took their blood samples extracted total RNA and prepared cDNA for gene expression using quantitative real time PCR analysis. We selected a number of genes from the insulin signaling pathway and some that had previously been shown to be related with type 2 diabetes [19]). Relative expression levels of Insulin receptor substrate 1 (INSR-1), PI4K, AKt, TSC2, NFκB, ICOS, LOC6 and Calg were analysed with real time PCR. Figure 4 shows that the differential expression patterns of these genes are consistent with a type 2 diabetes state. INSR, located upstream of the insulin pathway and a target of insulin, was downregulated hence possibly affecting the downstream events such as, PI4K, AKt and TSC2, Similarly immune regulators previously associated with insulin dysregulation such as NFκB, the proinflammatory cytokine mediator S100/Calgranulin and the inducible T-cell costimulator ICOS were down expressed in the HIV-R group compared to the HIV-Neg.

## Discussion

CD26/DPPIV is a multifunctional protein ubiquitously expressed in both soluble and cell surface forms in various endothelial and epithelial cells including T-cells and exert it different functions depending on cell type and conditions it is expressed [20]. It acts as a proteolytic enzyme, receptor and co-stimulatory protein and its substrates have been reported to be involved in various physiological process including immunomodulation and homeostasis. Dysregulation of CD26/DPPIV has been suggested to result in various pathophysiological process including rheumatoid arthritis, melanoma and Crohn's disease among others [20,21]. Its association with Type 2 diabetes is however well confirmed with DPPIV inhibitors being used worldwide as approved regimens for anti-type 2 diabetes treatment [22]. Our observation of high CD26/DPPIV expression, significant down expression of genes in the insulin signaling pathway and impaired fasting glucose state (IFG) implied an evolving type 2 diabetes condition among the HIV resistant commercial sex workers. The duration for evolution to full type 2 diabetes from an IFG state is however controversial. While some studies indicate that IFG conditions often lead to diabetes within 3 years, others have suggested a 50% risk over a ten year period [23]. The Nairobi female sex worker cohort has been followed up biannually for periods longer than ten years, and though blood sugar analysis has not been a routine feature, cases of overt Type 2 diabetes has rarely been observed. We postulate that this population, despite its high CD26/DPPIV levels may not be at risk of developing type 2 diabetes. There will however be a need to incorporate type 2 diabetes evaluations in future follow-up to determine the validity of this assertion taking into account specific DPPIV enzyme activity which we did not evaluate in this study.

Although an association of high CD26/DPPIV expression with HIV resistance is well supported by our data at the gene, soluble protein and cell surface level, the mechanism by which it protects against HIV acquisition remains unclear. One possibility is the key role it plays in cleaving off dipeptides from amino termini containing proline or alanine moieties at the penultimate position [24]. A number of chemokines have been shown to share this sequence at their termini including RANTES, an interleukin 8 superfamily chemokine has been shown to inhibit HIV infection by competing with the virus for its coreceptor CCR5. Previous data has shown that in contrast to intact RANTES, a DPPIV truncated RANTES inhibited in vitro HIV infection of mononuclear cells by M-tropic HIV strains fivefold efficiently [15,24]. Similarly several workers have shown that HIV infected subjects have defective CD4+ T cells with a poor ability to recognize and respond to antigens [25,26]. Antigen recognition is the preserve of CD4+ T-cells expressing CD26/DPPIV which proliferate in response to soluble antigens and activate MHC-restricted cytotoxic T cells resulting in annihilation of virally infected cells [20,27]. This has been supported by findings that antigen response in HIV-1 infected individuals could be restored by addition of soluble CD26/DPPIV in vitro, suggesting an important role for CD26/DPPIV in HIV-specific immunity [28].

The probable mechanism of CD26/DPPIV activity against HIV acquisition in the Nairobi cohort may however add a new dimension to the above observations. Our recent findings indicate a significantly lowered immune activation state among the HIV-R female sex workers as compared to HIV negative controls [13,29]. This lowered activation state or immune quiescence has also been observed among HIV exposed seronegative partners of HIV infected spouses [7] and HIV uninfected hemophiliacs transfused with HIV seropositive donor blood [30]. The contribution of CD26/DPPIV to a lowered immune activation state among the HIV-R female sex workers is puzzling.

There is substantial evidence that CD26/DPPIV can act as the trigger to immune activation [31-34]. This has been supported by studies showing that reversible DPPIV inhibitors suppress proliferation of human PBMCs s and enhance production of cytokines that inhibit antigen stimulation of T-cells [35]. Our findings of high CD26/DPPIV expression *in vivo *in an environment of lowered T-cell immune activity is hence intriguing. One possible explanation maybe due to the perturbations of the insulin signaling pathway in these women which induced the down expression of the nuclear factor kappa-light-chain-enhancer of activated B cells (NF-κB) and other pro-inflammatory enhancers (calgranulin and ICOS). NF-κB is a protein complex that regulates the expression of a multitude of immune response genes including cytokines, chemokines, antigen presenting cells and adhesion receptors [36]. In response to a variety of stimuli, including cytokines, viral and bacterial pathogens, a latent inactive NF-κB complex is activated in the cytoplasm through phosphorylation and translocates to the nucleus, where it stimulates transcription of genes. Activation of NFκB by the inflammatory cytokine alpha tumor necrosis factor (TNFα) has been shown to involve the phosphotidylinositol pathway together with its downstream targets-Akt, TSC1 and TSC2 through IRS 1 [37,38]. Quantitative real time PCR confirmed low expression in IRS1, PI4K TSC and, NFκB among the HIV resistant female sex workers (Figure 4). We hypothesize that in presence of high CD26/DPPIV, the reduced insulin signaling leads to a low activity of phosphatidylinositol cascade system which in turn resulted in a lower expression of TSC-2, a build-up of the MTORC1 and subsequent repression of NFκB. In-depth functional studies will be required to test this assertion. Our observations suggest that metabolic and signaling pathways that predispose to an impaired glucose fasting state and Type 2 diabetes may be new correlates of HIV resistance. In addition, it underscores the key role of a systems biology in discovery of novel candidate biological markers that may be crucial in the design of effective preventive strategies against HIV/AIDS.

## Methods

### Study population

The Nairobi (Pumwani) commercial sex worker cohort was established in 1985 and has provided vital data that there might be biological mediated resistance to HIV infection [11,39]. Despite repeated exposures to HIV-1, a number of women in the cohort have remained HIV uninfected for long periods and have epidemiologically been defined as resistant. The definition used in the current study were female sex workers actively followed in the cohort for more than seven years and persistently seronegative at biannual visits and negative by a sensitive PCR assay for proviral HIV-1 [8]. HIV infected commercial sex workers who were not on antiretroviral treatment (HIV+) and HIV negative antenatal clinic attendees (HIV Neg) were included as controls. Written Informed consent was sought from all volunteers and the study received ethical approval from ethical review boards of University of Nairobi and the Kenya Medical Research Institute.

### Soluble CD26/DPPIV analysis

Peripheral blood samples were randomly obtained from HIV Resistant and HIV positive female sex workers. Samples were also obtained from a HIV negative population of female antenatal clinic attendees matched for age (38.5 yrs) and race from the same part of Nairobi. All samples were separated to yield plasma and peripheral mononuclear cells. Soluble CD26/DPPIV plasma levels were quantified by Human DPPIV/CD26 Quantikine ELISA kit (R&D Systems, Minneapolis MN) following the manufacturer's recommendation. Briefly, standards and samples were diluted to a 1:1000 and ran in triplicate. Optical densities of the standards were used to generate a standard curve to determine sample concentration. Mean values were calculated and tests for significance determined with Graphpad Prism using student -t tests. A total of 300 subjects (100 in each arm) were used for the study.

### Cell surface CD26 expression analysis

To determine expression differences at cell surface level (CD26) a 3 color flow-cytometry (FACs) analysis was performed with the use of anti-CD26 antibody. PBMC were stained for CD4, CD8, and DPP4 washed and fixed using 1% paraformaldehyde. FACS data was analyzed using FlowJo (Tree Star, Ashland, OR). Lymphocytes were gated on forward versus side scatter, and then CD4 versus CD8. CD26/DPPIV expression levels were analyzed on CD4+ cells. Gates were set on the basis on single stained samples that did not contain CD26/DPPIV and used to calculate expression and mean florescent intensity levels. Significant values were determined in Prism using the Mann Whitney test.

### Investigation for clinical type 2 diabetes

To investigate clinical type 2 diabetes, commercial sex workers who had a significantly elevated soluble CD26/DPPIV plasma concentration were re-assessed for blood sugar levels. Identified volunteers were requested to suspend sex work for at least 24 hours and undergo an overnight fast. Blood samples were obtained before breakfast and analyzed for blood sugar using the glucose oxidase test system (Accu-check, Roche, Basel). Volunteers with blood glucose levels above 125 mg/dl (6.9 mmol/l) were considered diabetic and those between 100-125 mg/dl considered as having Impaired fasting glucose state as per the ADA definition criteria [18].

### Expression patterns of genes in the insulin/type 2 diabetes signaling pathway

Quantitative PCR analysis of selected genes in the insulin signaling pathway from a representative sample of HIV-R and HIV-Neg controls were done using Biosystems 7500 Real-Time PCR System (ABI, Foster City, CA). Assay protocols followed the Quantitect SYBR Green RTPCR a kit (Qiagen GmbH, Hilden DZ). Briefly 500 ng of total RNA extracted was reverse transcribed into cDNA using superscript II reverse transcript. Aliquots of equal amounts of cDNA from each sample were ran in duplicate and normalized using an internal control gene 18s RNA. Relative expression of each gene was determined from a standard curve generated from a pool of cDNA from the same samples. P-values for significance were analyzed using student t-test.

## Competing interests

The authors declare that they have no competing interests.

## Authors' contributions

EMS conceptualized, designed, supervised the study and did the manuscript write-up. BO, LM, MR, WA, EM and AM supported the laboratory analysis. JK and CW were project physicians involve in day to day sample collection, treatment and follow-ups. BlTB, ML, FP and SM were project advisors and contributed to the manuscript write-up. All authors have read and approved the final manuscript.

## References

[B1] Kallings LO The first postmordern epidemic: 25 years of HIV/AIDS J internal Med 2008 263 218 243 10.1111/j.1365-2796.2007.01910.x 18205765

[B2] Stover J Fidzani B Molomo BC Estimated HIV trends and program effects in Botswana PLos ONE 2008 3 e3729 10.1371/journal.pone.0003729 19008957 PMC2579326

[B3] Wainberg M Kuan-Te Jeang 25 years of HIV research-progress and perspectives BMC Medicine 2008 6 31 10.1186/1741-7015-6-31 18976462 PMC2585089

[B4] Saez-Cirion A Versmisse P Persistent resistance to HIV-1 infection in CD4 T cells from exposed uninfected Vietnamese individuals is mediated by entry and post entry blocks Retrovirology 2006 3 81 10.1186/1742-4690-3-81 17092330 PMC1636660

[B5] Rowland-Jones SL HIV specific cytotoxic T-cell activity in a HIV-exposed but uninfected infant Lancet 1993 341 860 861 10.1016/0140-6736(93)93063-7 8096564

[B6] Clerici M Sison AV Cellular immune factors associated with mother-to-infant transmission of HIV AIDS 1993 7 1427 1433 10.1097/00002030-199311000-00004 8280407

[B7] Begaud E Chartier L Reduced CD4 T-cell activation and *in vitro *susceptibility to HIV-1 infection in exposed uninfected Central Africans Retrovirology 2006 3 35 10.1186/1742-4690-3-35 16792805 PMC1524799

[B8] Fowke KR Nagelkerke NJ Resistance to HIV-1 infection among persistently seronegative prostitutes in Nairobi, Kenya Lancet 1996 348 1347 1351 10.1016/S0140-6736(95)12269-2 8918278

[B9] Liu R Paxton WA Homozygous defect in HIV-1 coreceptor accounts for resistance of some multiply exposed individuals in HIV-1 infection Cell 1996 86 367 377 10.1016/S0092-8674(00)80110-5 8756719

[B10] Rowland-Jones SJ Sutton J HIV specific cytotoxic T-cells in HIV exposed but uninfected Gambian Women Nat Med 1995 1 59 64 10.1038/nm0195-59 7584954

[B11] Horton RE Ball TB Cervical HIV-specific IgA in a population of commercial sex workers correlates with repeated exposure but not resistance to HIV AIDS Res and Hum Retroviruses 2009 25 83 92 10.1089/aid.2008.0207 19108692

[B12] Rowland-Jones SL Dong T Cytotoxic T-Cell responses to multiple conserved HIV epitopes in HIV-resistant prostitutes in Nairobi J Clin Invest 1998 102 1643 4 10.1172/JCI4314 9802890 PMC509124

[B13] Songok E Ball B Luo Ma Rono M Apidi W Kaefer N HIV Resistant commercial sex workers have a unique gene expression signature pattern Abstract no. 323, Keystone Symposium, Overcoming the Crisis of TB and HIV, Oct 20-25, 2009, Arusha Tanzania 2009

[B14] Rodrigues F Bone RF Best JMC Gillespie JW Post analysis follow-up and validation of microarray experiments Nature Genetics 2002 32 509 514 10.1038/ng1034 12454646

[B15] Morimoto C Lord C Zhang C Duke-Cohan JS Role of CD26/dipeptidyl peptidase IV in human immunodeficiency virus type 1 infection and apotosis Proc Natl Acad Sci 1994 91 9960 4 10.1073/pnas.91.21.9960 7937926 PMC44937

[B16] Mentlein R Gallwitz B Dipeptidyl peptidase IV hydrolyses gastric inhibitory polypeptide, glucagon like peptide 1 (7-36) amide peptide histidine methionine and is responsible for their degradation in human serum Eur J Biochem 1993 214 829 835 10.1111/j.1432-1033.1993.tb17986.x 8100523

[B17] World Health Organization Definition and Diagnosis of diabetes mellitus and intermediate hyperglycemia 2006 http://www.who.int/diabetes/publications/Definition%20and%20diagnosis%20of%20diabetes_new.pdf

[B18] American Diabetes Association Standards of Medical Care in Diabetes Diabetes Care 2006 29 suppl s4 s42 16373931

[B19] Prokopenko I McCarthy MI Lindgren CM Type 2 diabetes: new genes, new understanding Trends in Genetics 2008 24 613 621 10.1016/j.tig.2008.09.004 18952314 PMC7116807

[B20] Boonacker E Cornelis J Van Noordern F The multifunctional or moonlighting protein CD26/DPPIV European J Cell Biology 2003 82 53 73 10.1078/0171-9335-00302 12647932

[B21] Gorrell MD Dipeptidyl peptidase IV and related enzymes in cell biology and liver disorders Clin Sci 2005 108 277 92 10.1042/CS20040302 15584901

[B22] Ritcher B Bandeira-Echtler E Bergerhoff K Lerch C Emerging role of dipeptidyl pep tidase-4 inhibitors in the management of type 2 diabetes Vasc Health Risk Manag 2008 4 753 6 19065993 10.2147/vhrm.s1707 PMC2597770

[B23] Nichols GA Hillier TA Brown JB Progression from newly acquired impaired fasting glucose to type 2 diabetes Diabetes Care 2007 30 228 233 17259486 10.2337/dc06-1392 PMC1851903

[B24] Proost P De Meester I Schols D Struyf S Amino-terminal truncation of chemokines by CD26/dipeptidyl-peptidase IV-conversion of RANTES into a potent inhibitor of monocyte chemotaxis and HIV infection J Biol Chem 1998 273 7222 719 10.1074/jbc.273.13.7222 9516414

[B25] Blaquez MV Madueno JA Gonzalez R Jurado R Selective decrease of CD26 expression in T cells from HIV-1-infected individuals J Immunol 1992 149 3073 3077 1357035

[B26] Van Noesel CJ Gruters RA Terpstra FG Functional and phenotypic evidence for a selective loss of memory T cells in asymptomatic human immunodeficiency virus-infected men J Clin Invest 1990 86 293 9 10.1172/JCI114698 1694865 PMC296720

[B27] Dong RP Morimoto C Role of CD26 for CD4 memory T cell function and activation Hum Cell 1996 9 153 162 9183643

[B28] Hosono O Homma T Kobayashi H Munakata Y Decreased Dipeptidyl Peptidase IV Enzyme Activity of Plasma Soluble CD26 and Its Inverse Correlation with HIV-1 RNA in HIV-1 Infected Individuals Clinical Immunology 1999 91 283 295 10.1006/clim.1999.4711 10370373

[B29] Cohen J HIV Natural Resistance Field finally overcomes resistance Science 2009 326 1477 10.1126/science.326.5959.1476 20007880

[B30] Salkowitz JR Purvis SF Meyerson H Zimmerman P Characterization of High-Risk HIV-1 seronegative hemophiliacs Clinical Immunology 2001 98 200 211 10.1006/clim.2000.4969 11161976

[B31] Ohtsuki T Tsuda H Morimoto C Good or Evil: CD26 and HIV Infection J Dermatol Sci 2000 22 152 60 10.1016/S0923-1811(99)00081-X 10698152

[B32] Reinhold D Gohil A Wrenger S Reinhold A Review: Role of dipeptidyl peptidase IV (DP IV)-like enzymes in T lymphocyte activation: investigations in DP IV/CD26-knockout mice Clin Chem Lab Med 2009 47 268 74 10.1515/CCLM.2009.062 19676138

[B33] Ohnuma K Takahashi N Yamochi T Role of CD26/dipeptidyl peptidase IV in human T cell activation and function Front Biosci 2008 13 2299 310 10.2741/2844 17981712

[B34] Schon E Dipeptidyl peptidase IV, a membrane enzyme involved in proliferation of T-lymphocytes Biomed.Biochim Acta 1985 44 K9 K15 2860900

[B35] Reinhold D Inhibitors of dipepetidyl peptidase 4 induce secretion of transforming growth factor β1 in PWM-stimulated PBMC and T-cells Immunology 1997 91 354 360 10.1046/j.1365-2567.1997.d01-2258.x 9301523 PMC1364003

[B36] Hiscott J Kwon H Genin P Hostile takeovers: viral appropriation of the NF-KB pathway J Clin Invest 2001 107 143 151 10.1172/JCI11918 11160127 PMC199181

[B37] Ozes ON Mayo LD Gustin JA Pfeffer SR NF-kappaB activation by tumour necrosis factor requires the Akt serine-threonine kinase Nature 1999 401 82 5 10.1038/43466 10485710

[B38] Ghosh S Tergaonkar V Rothlin CV Essential role of tuberous sclerosis genes TSC1 and TSC2 in NF-kappaB activation and cell survival Cancer Cell 2006 10 215 26 10.1016/j.ccr.2006.08.007 16959613

[B39] Plummer FA Ball TB Resistance to HIV Infection among highly exposed sex workers in Nairobi: what mediates protection and why does it develop? Immunol Letters 1999 66 27 34 10.1016/S0165-2478(98)00182-5 10203031

